# A method for measuring individual research productivity in hospitals: development and feasibility

**DOI:** 10.1186/s12913-015-1130-7

**Published:** 2015-10-14

**Authors:** Caterina Caminiti, Elisa Iezzi, Caterina Ghetti, Gianluigi De’ Angelis, Carlo Ferrari

**Affiliations:** Research and Innovation Unit, University Hospital of Parma, Parma, Italy; Medical Physics Unit, University Hospital of Parma, Parma, Italy; Gastroenterology Unit, University Hospital of Parma, Parma, Italy; Infectious Diseases and Hepatology Unit, University Hospital of Parma, Parma, Italy

**Keywords:** Research productivity, Research output, Capacity building, Health care providers, Productivity measurement, Research institutions, University hospitals, Italy

## Abstract

**Background:**

Research capacity is a prerequisite for any health care institution intending to provide high-quality care, yet, few clinicians engage in research, and their work is rarely recognized. To make research an institutional activity, it could be helpful to measure health care professionals’ research performance. However, a comprehensive approach to do this is lacking.

**Methods:**

We conducted a literature analysis to determine how best to assess research performance. Our method was not restricted to bibliometric and citation parameters, as is usually the case, but also including “hidden” activities, generally not considered in research performance evaluations.

**Results:**

A set of 12 easily retrievable indicators was used and corresponding points assigned according to a weighting system intended to reflect the effort estimated to perform each activity. We observed a highly skewed score distribution, with a minority of health care professionals performing well across the indicators. The highest score was recorded for scientific papers (768/1098 points, 70 %). Twenty percent of researchers at our institution generated 50 % of points.

**Conclusions:**

We develop a simple method for measuring research performance, which could be rapidly implemented in health care institutions. It is hoped that the proposed method might be useful for promoting research and guiding resource allocation, although further evaluations are needed to confirm the method’s utility.

**Electronic supplementary material:**

The online version of this article (doi:10.1186/s12913-015-1130-7) contains supplementary material, which is available to authorized users.

## Background

It is widely accepted that research plays an essential role in developing new health care services and improving healthcare quality. Research provides new knowledge that can be transferred into practice, helps create advanced care environments, that attract the best physicians contributes to learning among young Health Care Professionals (HCPs) and ensures continuous education among established professional. In fact, hospitals engaged in research have been recognized to as providing better patient care. Therfore, adequate research capacity is a prerequisite for any public health care system striving to provide high-quality care [1].

The goal of evidence-based practice increasingly requires research to be embedded within the health care setting, making clinician participation an essential component of its success. In fact, clinicians are well-placed to identify relevant research ideas, design and conduct innovative projects, ensure translation of research into improved health outcomes, and solicit patient enrollment in experimental trials. Nevertheless, the international literature shows that only a minority of clinicians participate in research aproblem common to many countries [2–5].

This issue is particularly relevant in teaching hospitals, which have a responsibility to provide leadership in conducting, supporting and supervising research [5]. There is no standardized method for measuring HCP’s research efforts and their results; this is a key obstacle to the incorporation of research in hospitals as an institutional activity [6]. Such a method would, among other benefits, inform resource allocation decisions, encourage research participation among increasingly busy clinicians, and create accountability to the community for research projects.

To this end, we developed a mechanism that attempts to measure as objectively as possible research productivity, and tested it at our institution to determine its feasibility and utility. In this study, research productivity is defined as the product of research activities. The terms “research productivity”, “output” and “performance”, are used interchangeably.

## Methods

### Setting

The University Hospital of Parma is a large health care facility located in Emilia-Romagna, a region in northern Italy with a population of 5 million served by four university hospitals, four health care research institutes and 12 community hospitals.

Since 2004, regional legislation formally identifies research as a fundamental institutional activity, equal to patient care and continuous training. This policy underlies several funding initiatives aimed to promote research, with special attention to young professionals [7]. Regional hospitals are required proactively support their researchers, through clinical governance actions aiming to track research activities already underway, identify priority areas for resource allocation and infrastructure, and provide adequate tracking and recognition of researcher efforts. However, no method for measuring research has been devised and implemented across health care institutions in this region, where hospital productivity is currently only being measured in terms of patient care activity.

### Objectives

This work pursues the following objectives:

1. develop a simple method to measure individual research productivity and analyze hospital department performance
2. determine its feasibility and describe its potential usefulness in a large University Hospital

### Choice of indicator variables

Literature analysis was conducted to determine how best to assess research performance. The terms “research output”, “research productivity”, and “research performance” were used to retrieve potentially relevant studies published over the last 5 years. Articles that only analyzed bibliometric indices and those that did not report on an empirical setting were not considered.

Although the evaluation of research productivity would ideally include the assessment of impact, in practice this is extremely difficult to achieve, because the multifaceted nature of evaluation, the lack of standard terminology, and the heterogeneity of empirical experiences make it hard to identify a preferred model of impact measurement [8]. For this reason, the measure of research output is often used as a proxy for impact. A wide range of indicators and metrics are available for this purpose, and their choice depends on considerations of their strengths and limitations [8]. The most widely used research output indicators are bibliometric and citation parameters (e.g. number of publications in peer-reviewed journals, impact factor and H-index) [9]. These are very simple to calculate, but also exhibit various limitations that have been extensively described [9–11].

Wootton [12] recently proposed a simple method to measure research output, defining an indicator simple enough to be calculated and generalizable to other settings, but still able to capture the complexity of research productivity. The indicator, inspired by the analysis of 12 reports on research productivity, is constructed on the following three domains, based on data relating to individual researchers: (i) research grant income, (ii) peer-reviewed publications and (iii) PhD student supervision. Activity in each domain is converted to points, which are used to calculate a score for research output that allows comparisons: (a) within an organizational unit, for example from year to year, or (b) in the same year between organizational units (e.g. research teams, wards, departments, and hospitals). The proposed score was arbitrary, because no validated and widely accepted metrics exists, but it was compared with an independent assessment made by a group of expert researchers, which yielded a significant correlation of 71 %.

This indicator, however, neglects a range of “hidden” activities that are also relevant to research output assessment. There are described in a well-known editorial by former BMJ Editor Richard Smith [13]. These include, for example, participation in the preparation of guidelines, teaching activities in the field of research, and peer-reviewing.

In another interesting work- by Mezrich et al. [14] reported the development of a more complex system for the assessment of the productivity of a ward/academic department, which assigns points to research activities by considering the estimated effort required to perform each activity and its attributed academic value.

## Results

### Development of the method

The approach we defined is an adaptation of the model proposed by Wootton [12], integrated with other types of activities indicated by Smith [13] and inspired by the metrics used by Mezrich et al. [14]. The choice of research activities was determined by the availability of required information. The weighting system was constructed considering the hypothesized effort for all indicators,. For some indicators, specific criteria were also applied.

For each HCP, a set of information easily retrievable from existing administrative sources (mostly the Parma Ethics Committee’s archive) and from bibliographic databases (e.g.,ISI Web of Knowledge) was collected. These includedcompetitive research funding, publications, students/collaborators supervised, commissioned studies and patent filing. Additionally, some information usually not recorded was gathered by means of a simple questionnaire adapted from the literature, a tool used by German researchers for the measurement of the effects of a training program designed to improve HCPs’ research skills [15]. This adapted questionnaire (see Additional file 1) has been employed at our institution since 2012 to gather data on self-reported participation in research activities [16] and contains the remaining seven indicators used in this study. To be included in the final score, research activities indicated in the questionnaire had have been previously documented.

The set of proposed indicators, the weighting system and the number of possible points assigned to each are depicted in Table 1.

**Table 1:** Indicators for the quantification of research activity and attributed values

Indicator	Criteria for point assignment	Weighting based on	Scaling factor used
Grants	- Obtained competitively		€24.000^a^= 1 point
	- Referring to all or part of the year in question		
Publications	- Listed in ISI Web of knowledge (peer-reviewed)	- Position/role of author in the project	1 paper = 1 point* position value* Normalized IF
	- Published in the year in question	- Normalized journal impact factor	
	- Abstracts are excluded		
PhD students/External collaborators	- Financed by research grants not obtained in the framework of competitive programs	Time dedicated to supervision activities	1 PhD student, collaborator/year =1 point
	- Supervisor during all or part of the year in question		
Projects	- Not financed with funds from competitive programs		1 project= 0.2 if spontaneous study; 0.1 if commissioned study
	- Starting in the year in question		
	- Principal Investigator		
Patent filing	- Registration in the year in question		1 patent= 0.1 points
Training in the field of research methodology	- Documented attendance in the year in question		1 master= 0.2 points 1 course= 0.1 points
Research proposals	Submitted to competitive programs but not awarded funding in the year in question		1 protocol=0.1 points
Member of committee for guideline production	Documented participation in the year in question		1 guideline= 0.1 if national; 0.2 if international
Teaching activity on own research	Documented in the year in question		teaching activity= 0.2 points
Serving as peer-reviewer (of grants and papers)	Documented in the year in question		1 journal/grant= 0.2 points if international; 0.1 points if national
Abstracts, articles, book chapters, etc.	- Not peer-reviewed		1 document= 0.2 if available internationally; 0.1 if available nationally; 0.05 if abstract
	- Published and retrievable in the year in question		
Article submission to peer-reviewed journals	- Not accepted for publication in the year in question		1 paper= 0.1 points
	- Documented		

^a^Lowest award for a standard Italian research grant (Decreto MIUR 9/3/2011 n.102), chosen as scaling factor to allow comparison of this indicator between countries [Wootton]

For the first indicator, concerning grants acquired by the Principal Investigator in competitive research funding programs, one point is assigned for every €24.000 awarded; this is the lowest award for a standard Italian research grant (Decree of the Italian Ministry of Education, Universities and Research, no. 102, 9 March 2011). This solution is suggested by Wootton as a scaling factor to facilitate comparisons between countries. Thus because on average the annual cost of a resident physician is three times greater than that of a research grant, one point is assumed to reflect about 1/3 of an HCP’s annual work (approximately 4 months). This assumption was used to assign scores taking -into account the estimated time required to carry out a given research activity relative to others.

For the publication indicator, each paper received a score weighted by the Normalized journal Impact Factor (NIF) and by author position (Table 2). The NIF is an adjusted method for calculating the impact factor that takes into account the diversity of citing behavior in different disciplines and is inteded intended to assess the relative position of journals, potential employers, and researchers within each field [17]. Weighting criteria for the publication score are based on the method developed by Tscharntke [18], whereby the first author is awarded the highest value, but the second and last authors also receive a higher score than the other coauthors.

**Table 2:** Scheme for assigning credit to authors of multiauthor

No. of authors	First author	Second	Third	Fourth	Fifth	Sixth	Seventh	Eight	Ninth	Tenth	Total
1	1,00										1,00
2	0,70	0,30									1,00
3	0,50	0,25	0,25								1,00
4	0,50	0,20	0,10	0,20							1,00
5	0,40	0,20	0,10	0,10	0,20						1,00
6	0,40	0,20	0,07	0,07	0,07	0,20					1,00
7	0,40	0,20	0,05	0,05	0,05	0,05	0,20				1,00
8	0,40	0,20	0,04	0,04	0,04	0,04	0,04	0,20			1,00
9	0,40	0,20	0,03	0,03	0,03	0,03	0,03	0,03	0,20		1,00
10	0,40	0,20	0,03	0,03	0,03	0,03	0,03	0,03	0,03	0,20	1,00

For the remaining indicators, score assignment was straightforward, as shown in Table 1. The calculation performed for this set of indicators allows us to obtain (for each HCP) a combined score resulting from the sum of non-dimensional values, which permits spatial and temporal comparisons.

### Implementation

Overall, the time needed to create a single database, process indicators for each HCP and analyze data was about 9 weeks of work by one person. The analysis was performed using SAS version 8.2. Time for data collection and analysis may be significantly reduced with the use of web-based software into which pertinent data may be entered by HCPs themselves.

To allow for comparisons with other institutions, wards were grouped into the following six areas, which represent relatively homogeneous research activities: Surgery units; Diagnostic Services; Emergency Medicine; General Medicine, Geriatrics and Rehabilitation; Specialized Medicine; Pediatrics and Gynecology. To reduce variability (Coefficient of Variation = 38 %) due the different numbers of HCPs in each area, estimates were corrected by direct standardization. For each area, along with the sum and the weighted sum, the mean score (per capita output) and corresponding range are also provided.

### Intra- and inter comparisons

Tables 3 and 4 summarize respectively raw data and calculated values for research activity relating to the year 2013, subdivided for each indicator. The most relevant findings with respect to this work’s objectives are the following:

**Table 3:** Research activity for indicators – Raw data year 2013

Area description	Grants of competitive programs	Projects with grants	Publications	PhD students/external collaborators	Projects not funded by competitive programs	Patent filing	Training	Research proposals	Member of committee for guideline production	Teaching activity in the field of research	Peer-reviewing	Abstract, articles, books, etc.	Article submission to peer-reviewed journals
Surgery units	€ 128.625	5	69	0	9	0	5	16	6	9	19	9	7
Diagnostic Services	€ 250.368	2	184	12	3	0	5	14	9	5	9	6	6
Emergency medicine	€ 61.175	1	65	0	1	0	4	3	4	2	6	1	0
General medicine, Geriatrics and rehabilitation	€ 56.458	5	42	1	10	0	5	15	6	3	6	5	5
Specialized medicine	€ 3.251.828	17	198	33	78	1	10	34	15	30	40	14	17
Pediatrics and gynecology	€ 129.725	2	39	0	7	0	2	7	5	2	7	4	1
TOTAL (% of row)	€ 3.878.179	35 (3 %)	597 (51 %)	46 (4 %)	108 (9 %)	1 (0 %)	31 (3 %)	89 (8 %)	45 (4 %)	51 (4 %)	87 (7 %)	39 (3 %)	36 (3 %)
Area description	No. of employees	Grants	Publications	PhD students/external collaborators	Projects not funded by competitive programs	Patent filing	Training	Research proposals	Member of committee for guideline production	Teaching activity on own research	Peer-reviewing	Abstract, articles, books, etc.	Article submission to peer-reviewed journals
Surgery units	152	5.4	98.0	0	1.5	0	1.1	2.1	0.8	3.4	8.3	1.2	1.5
Diagnostic services	237	10.4	258.0	12	0.6	0	0.7	2	1.1	1.6	6.4	1.1	1.2
Emergency medicine	148	2.5	0.0	0	0.2	0	0.2	0	0	0	0	0	0
General medicine, geriatrics and rehabilitation	111	2.4	46.7	1	1.7	0	0.7	0.4	0.6	0.6	1.7	0.1	0
Specialized medicine	179	135.5	317.5	33	10.3	0.1	2	9.3	3.3	10.6	27.8	2.1	4.5
Pediatrics and gynecology	69	5.4	47.5	0	1.1	0	0.2	1.3	0.7	1.4	6.1	0.7	0.3
TOTAL (% of row)	896	161.6 (15 %)	767.7 (70 %)	46 (4 %)	15.4 (1 %)	0.1 (0 %)	4.9 (0 %)	15.1 (1 %)	6.5 (1 %)	17.6 (2 %)	50.3 (5 %)	5.2 (0 %)	7.5 (1 %)

**Table 4:** Research activity for indicators – Attributed values year 2013

Area description	No. of HCPs	Sum [°]	Weighted Sum [§]	Per capita output (mean)	Range (min-max)
Surgery units	152	123.2	20.9	0.8	(0–6)
Diagnostic services	237	295.1	78.1	1.2	(0–93)
Emergency medicine	148	2.9	0.5	0.4	(0–15)
General medicine, geriatrics and rehabilitation	111	55.9	6.9	1.5	(0–16)
Specialized medicine	179	556.0	111.1	2.2	(0–59)
Pediatrics and gynecology	69	64.7	5.0	0.9	(0–6)
TOTAL	896	1097.9	222.4	1.2	(0–93)

[°]:Sum of attributed values with scaling factor – [§]: Weighted for No. of HCPs on total HCPs

- When no score is assigned, prevailing activities are publications (597/1165, 51 %), projects not funded by competitive programs (108/1165, 9 %) and research proposals submitted to competitive programs but non awarded (89/1165, 8 %).
- The highest score was recorded for scientific papers (768/1098 points, 70 %), followed by research grant income (15 %) and peer-reviewing (5 %). Together, these three items account for 90 % of the research output at our institution.
- The area of specialized medicine exhibited the highest research productivity, even after standardization (111/222). This means that a mere 20 % of HCPs at our institution produced 50 % of points
- The annual mean per-capita output score was 1.2 points, ranging from 0.4 to 2.2 points (indicated the the highest-scoring researchers were nearly six times more productive than lowest scoring researchers).

**Fig. 1:**
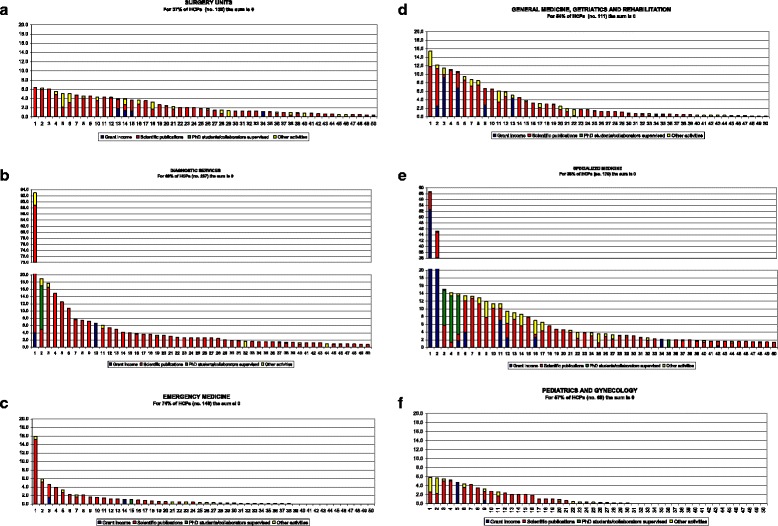
**a** (Research output for each HCP belonging to the area) – SURGERY UNITS. **b** (Research output for each HCP belonging to the area) – DIAGNOSTIC SERVICES. **c** (Research output for each HCP belonging to the area) – EMERGENCY MEDICINE. **d** (Research output for each HCP belonging to the area) – GENERAL MEDICINE, GERIATRICS AND REHABILITATION. **e** (Research output for each HCP belonging to the area) – SPECIALIZED MEDICINE. **f** (Research output for each HCP belonging to the area) – PEDIATRICS AND GYNECOLOGY

Figure 1 shows the research output for individual HCPs belonging to each area, for the following indicators: grant income, scientific publications, PhD students/collaborators supervised, and other activities. Our analysis indicated:

- Within all areas, few individuals obtained high scores, whereas the majority received low scores or zero points
- Only a few individuals performed well across multiple indicators, whereas for the majority, output mainly consisted of publications.

**Fig. 2:**
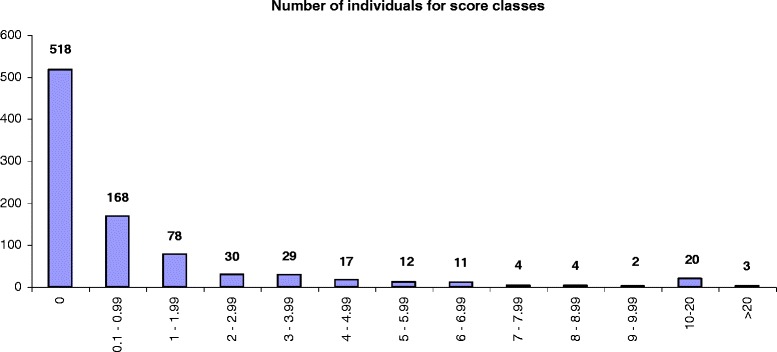
Score frequency distribution

Figure 2 summarizes the individual score distribution for score classes, which shows even more clearly that high research productivity was only achieved by a small group of HCPs.

## Discussion

We present a novel method for measuring research performance. The results obtained by our method’s implementation at a large Italian University Hospital highlight the simplicity of its implementation and describe its potential uses.

To our knowledge, this is the first comprehensive approach to measuring individual research output in hospitals that also includes “hidden” research activities, which are essential to ensure high-quality patient care, such as participation in the definition of guidelines, submission of research proposals to competitive funding programs regardless of funding acquisition, and teaching activities concerning one’s own research. This system exhibits many potential strengths and possible applications: it enables identification of identify which HCPs are highly productive in research, reveals of areas potentially in need of improvement, and provides indications for resource allocation.

Our work differs from Wootton’s study in many respects. First other things, implementation lasted 1 year and involved the entire institution, whereas for Wootton’s study lased 5 years and concerned two departments. Still, the two studies are similar enough to make a direct comparison of results. In both studies, score distribution is considerably skewed, and most points are earned by a small number of HCPs, mostly performing well on the publication indicator.

The chosen indicators and attributed scores still remain to be validated and widely shared. In fact, as evident in Tables 3 and 4, the chosen weighting system leads to the dominance of publication output and grant income. Other hospitals may feel that a more balanced scorecard would be preferable. However, validation was not the aim of this work, also because a precise use of results has not yet been defined. In fact, as Mezrich et al. pointed out by [14], for some purposes, such as measuring change in activity or productivity from one year to the next or the relative productivity of individuals performing similar activities in a single division or at different institutions, the values chosen would not matter, as long as they were consistent for all HCPs. A validated weighting system may instead be used as a tool to guide and promote research. For instance, more points may be assigned to strategic research activities (e.g., supervision of young PhD students and research collaborators), or rankings may be used (e.g., reviewers for prestigious international journals could be awarded higher scores). However, such systems should be applied with caution, as pointed out in a recent systematic review [19] on the effects of strategies introduced in academic medical centers to assess productivity as part of compensation schemes. The results of the 9 study review demonstrate that these strategies improve research output and help to achieve the department’s mission, but may have unintended negative consequences; for instance, HCPs may assume that items not included in the evaluation are less important and may thus neglect them.

It must be emphasized that this study is based on secondary data not collected for the purpose of this research, which may have led to an underestimation of the score, particularly concerning “hidden” activities, which had to have been previously documented by HCPs.

## Conclusions

Although further evaluations is needed, this work suggests that the proposed method may is feasible and may be useful to achieve different purposes, such as:

- Guiding funding of health care facilities, as is done with patient care (for instance through Diagnosis-Related Groups - DRGs)
- Including research activity in the assessment of a ward’s productivity, in the analysis of the workload and in subsequent allocation of necessary resources
- Overcoming the current disparity observed in Italian university hospitals, where recognition for research activities is ensured to HCPs employed by the university but not to those employed by the hospital, though both groups work in the same institution
- Highlighting the most productive and authoritative research centers, which may be qualified as centers of excellence for research worth being supported and enhanced
- Providing information that could form the basis for a regional research network, according to a Hub and Spoke model, to increase research capacity in facilities that do not have research as their mission and to prevent study duplication and consequent waste of resources.

## Additional file

Additional file 1:
**Questionnaire to record participation in resarch activities.** (DOC 32 kb)

## Abbreviations

HCP: Health-Care Professionals
NIF: Normalized journal impact factor
DRG: Diagnosis-Related Groups
