# Supplementary material for: A method for measuring individual research productivity in hospitals: development and feasibility
Source: BMC Health Serv Res. 2015 Oct 14;15:468. doi: 10.1186/s12913-015-1130-7 (PMC4607003; doi:10.1186/s12913-015-1130-7)
Supplement: Additional file 1: — Questionnaire to record participation in resarch activities. (DOC 32 kb) [file 12913_2015_1130_MOESM1_ESM.doc]

#### PARTICIPATION IN RESEARCH ACTIVITIES

**Year 2013**

*The questionnaire is to be completed individually by each Health Care Professional who should indicate their own activities in the field of research carried out during 2013; mandatory fields are marked with a *. Questionnaires shall be sent online within March 2014.*

### Personal information

| Last name * |  |
| --- | --- |
| First name * |  |
| e-mail |  |
| Unit/Department | *Select ...* |

# ACTIVITIES

**[1] Did you attend specific training (courses/Master programs) in the field of research methodology? ***

🔾 NO

🔾 YES

## If YES

## Please specify _______________________________________________________________________

**[2] Did you contribute to the definition of study protocols or research proposals (eg. Grant applications) which were not initiated?** *

🔾 NO

🔾 YES

## If YES

## Please specify _______________________________________________________________________

**[3] Did you contribute to the drafting of scientific guidelines?** *

🔾 NO

🔾 YES

## If YES

## Please specify _______________________________________________________________________

**[4] Did you carry out teaching activities concerning research you were conducting?** *

🔾 NO

🔾 YES

## If YES

## Please specify _______________________________________________________________________

**[5] Did you act as a referee/peer reviewer?** *

🔾 NO

🔾 YES

## If YES

## Please specify _______________________________________________________________________

**[6] Did you author, or contributed to, books in the medical/scientific field?** *

🔾 NO

🔾 YES

## If YES

## Please specify _______________________________________________________________________

**[7 ] Did you submit papers to peer-reviewed journals, which were not accepted for publication?** *

🔾 NO

🔾 YES

## If YES

## Please specify _______________________________________________________________________
